# Degradable Organically-Derivatized Polyoxometalate with Enhanced Activity against Glioblastoma Cell Line

**DOI:** 10.1038/srep33529

**Published:** 2016-09-23

**Authors:** Shan She, Shengtai Bian, Ruichao Huo, Kun Chen, Zehuan Huang, Jiangwei Zhang, Jian Hao, Yongge Wei

**Affiliations:** 1 Department of Chemistry, Tsinghua University, Beijing 100084, China; 2 State Key Laboratory of Natural and Biomimetic Drugs, Peking University, Beijing 100191, China; 3 Department of Biomedical Engineering, School of Medicine, Tsinghua University, Beijing 100084, China; 4 Laboratory Animal Center, Fourth Military Medical University, Xi’an 710032, China

## Abstract

High efficacy and low toxicity are critical for cancer treatment. Polyoxometalates (POMs) have been reported as potential candidates for cancer therapy. On accounts of the slow clearance of POMs, leading to long-term toxicity, the clinical application of POMs in cancer treatment is restricted. To address this problem, a degradable organoimido derivative of hexamolybdate is developed by modifying it with a cleavable organic group, leading to its degradation. Of note, this derivative exhibits favourable pharmacodynamics towards human malignant glioma cell (U251), the ability to penetrate across blood brain barrier and low toxicity towards rat pheochromocytoma cell (PC12). This line of research develops an effective POM-based agent for glioblastoma inhibition and will pave a new way to construct degradable anticancer agents for clinical cancer therapy.

Polyoxometalates (POMs) are a large family of metal oxide cluster anions with different applications in magnetics^1^,^2^,^3^,^4^, material^5^,^6^,^7^,^8^,^9^, catalysis^10^,^11^,^12^,^13^, and medicine^14^,^15^,^16^,^17^,^18^,^19^,^20^,^21^,^22^,^23^,^24^,^25^. Among them, the medicinal chemistry of POM is of great interest because various POMs can exhibit effective anticancer^15^,^16^,^17^, antiviral^18^,^19^ and antibacterial^20^,^21^,^22^ performances by inducting cell apoptosis and inhibiting ATP generation^15^. Recently, many research works have revealed that a variety of POMs are capable of penetrating across the blood brain barrier (BBB)^23^,^24^,^25^, endowing POMs with opportunity to be available agents for brain diseases. Glioblastoma is the most common and primary malignant brain tumor, which possesses poor prognosis with <3% survival rate after 5 years of diagnosis^26^,^27^,^28^. However, currently used chemotherapy agent called temozolomide (TMZ) exhibits moderate pharmacodynamics and resistance problem^29^,^30^. Although many efforts have been devoted, it remains a challenge to develop new agents for glioblastoma’s treatment. POMs may be a promising candidate for glioblastoma’s treatment.

In the medicinal chemistry of POMs, previous works on cancer therapy were mainly focused on the heteropolyacids, including Keggin^14^,^19^,^20^,^21^ and Wells-Dawson^23^,^24^,^25^. However, these POMs are stable *in vivo* and cannot be timely excreted, causing the side effect. Because the slow clearance of POMs can interfere the body’s metabolism and result in the long-term toxicity^31^,^32^, which impedes the clinical application of POMs in cancer therapy. According to previous works on POMs, the Lindqvist-type hexamolybdate anion can be functionalized with various organic groups *via* covalent modification^33^,^34^,^35^,^36^,^37^. It is feasible to modify the hexamolybdate core with a cleavable group, which is metastable in the environment of cell incubation. Since the Mo ≡ N bond formed from imidolyization is active, the imidolyization may be one of the suitable choices^33^. Also, the synergistic effect between the organic moiety and POM cluster may promote the inhibitory performance towards cancer cells^16^,^38^.

Herein, we report our recent finding that a degradable organically-derivatized POM, named [Mo_6_O_18_(≡NC_6_H_4_-2-CH_3_-6-CON(Cy)-CO-NH-Cy)]^2−^ (POM-AMB-acy) (Fig. 1) can not only exhibit inhibitory performance towards malignant glioma cell (U251), but also cross the blood brain barrier (BBB), which is the key step to develop a practical agent for glioblastoma’s treatment^39^. This compound is consisted of one hexamolybdates moiety (POM) as the efficacious center and one N-acylureido group (acy) for degradation, linked by a 2-amino-3-methylbenzoxyl group (AMB), which may benefit to its efficacy. As acy group could be cleaved in the environment of cell incubation^40^ then destabilize the whole agent, POM-AMB-acy would be degraded, and eventually transformed into [MoO_4_]^2−^, which is the most common and easily-excreted form of the molybdenum element in human body^41^. In this way, a degradable POM-based compound may be developed as a promising candidate for glioblastoma inhibition with degradability.

## Results and Discussion

### Synthesis and characterization

**Figure 2:**
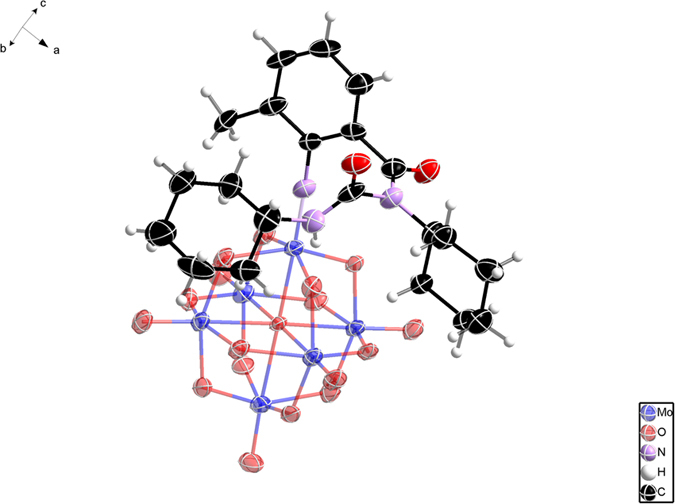
The molecular structure of POM-AMB-acy. Thermal ellipsoids are drawn at the 50% probability level.

A refluxing reaction of [Bu_4_N]_2_[Mo_6_O_19_], 2-amino-3-methylbenzoic acid and *N,N′*-dicyclohexylcarbodiimide (DCC) in dry acetonitrile can afford POM-AMB-acy in 23% yield after 20 h, as monitored by ESI-MS (Figure S1). The molecular structure of POM-AMB-acy has been clearly confirmed by single-crystal X-ray diffraction analysis (Fig. 2): its hexamolybdate cage is connected to the aromatic ring of 2-amino-3-methylbenzoxyl group *via* a Mo ≡ N triple bond with the Mo1-N1 bond length of 1.735 (4) Å and the linear C1-N1-Mo1 bond angle of 176.3 (8)°, which are in great agreement with the typical organoimido groups grafted at an octahedral d^0^ metal center^33^,^38^. Thus, the proposed POM-AMB-acy has been successfully fabricated.

### Inhibitory effect of POM against glioblastoma cell line

**Figure 3:**
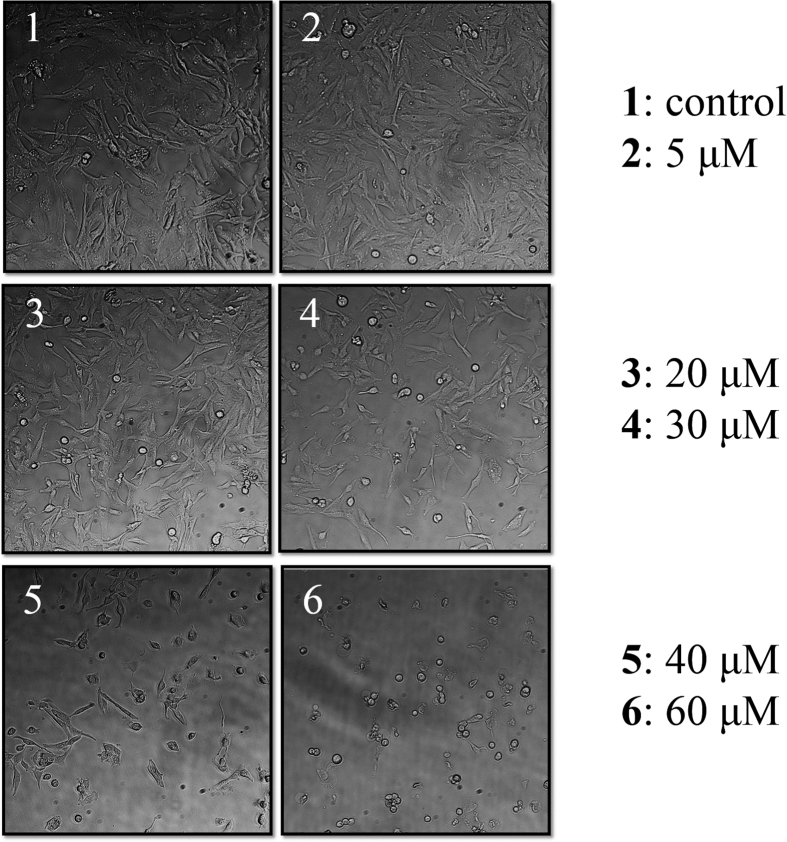
Morphology of U251 cells. The changes of U251 morphology at different concentrations of POM-AMB-acy treatment for 24 hours, examined by light microscopy. Cells were highly dense, had a spindle shaped body and acquired neuron-like morphology in the control group (1); Some cells showed spherical shapes (2); The number of the round shaped cells increased (3); Nearly half of the cells showed shrinkage and spherical shapes (4); Some cells underwent fragmentation (5); The majority of the cells apoptosis (6).

To understand whether POM-AMB-acy is indeed critical to glioblastoma’s inhibition, the proliferation and morphology of malignant glioma cells treated with POM-AMB-acy was studied. For demonstration, U251 cells were chosen to be utilized in this model research. As shown in Table 1, IC_50_ value for POM-AMB-acy is only 24.8 μM, while that for TMZ, the clinically-used key therapeutic agent towards malignant gliomas, is around 500 μM^29^,^42^. Moreover, examined by light microscopy (Fig. 3), U251 cells treated with 30.0 μM POM-AMB-acy showed shrinkage, loss of neuritis, swelling of cell bodies, and a global disruption of the dendritic networks, in contrast to the control group. Therefore, this POM-based anticancer agent can stimulate apoptosis of malignant glioma cells indeed.

It needs to be figure out whether the synergistic effect originates from POM and organic functional moiety can indeed facilitate the inhibition performance of POM-AMB-acy towards U251 cells. Recently, it is reported that an organic functionalized POM derived from amantadine exhibited better anticancer performance against cancer cells than non-substituted hexamolybdates or amantadine independently^16^ which suggested that the synergistic effect between POM and bioactive moiety can promote the inhibitory performance of POM-based agent towards cancer cells. In this research, the inhibitory effect between Na_2_MoO_4_, [Bu_4_N]_2_[Mo_6_O_19_], (Bu_4_N)_2_[Mo_6_O_18_(NC_10_H_15_)] (**POM-Ad**)^16^ and POM-AMB-acy towards U251 cell were evaluated. As shown in Table 1, the IC_50_ values of ‘6 equals of Na_2_MoO_4_·2H_2_O’ (53.4 μM) was two-folds higher compared to POM-AMB-acy (24.8 μM). This indicated that the functionalized POM, modified with *N-*acylureido group (acy) and 2-amino-3-methylbenzoxyl group (AMB), has better performance than metal ion itself. Furthermore, the difference between [Bu_4_N]_2_[Mo_6_O_19_], POM-Ad, and POM-AMB-acy suggested that imidoylization can improve the inhibitory performance of POM towards malignant glioma cells on certain degree.

**Figure 4:**
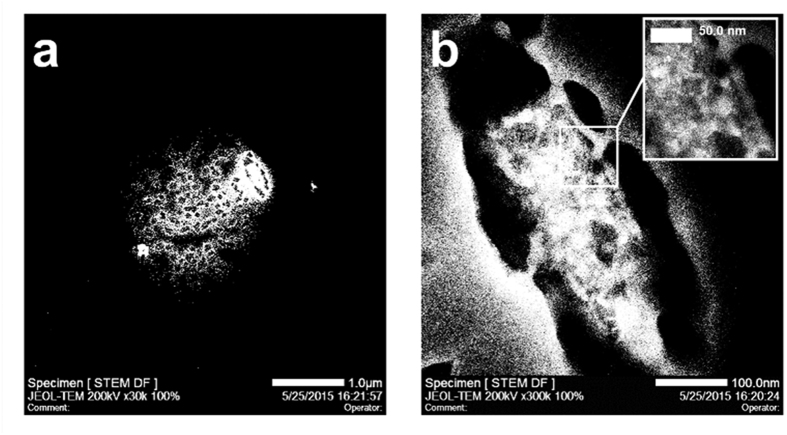
*In vitro* view of POM-AMB-acy towards U251 cells. (**a**) STEM image for molybdenum distribution in U251 cells treated with POM-AMB-acy. Scale bar = 1 μm. (**b**) Higher magnification of shinny bright location in Fig. 4a. Scale bar = 100 nm (inset: corresponding magnified image).

To investigate the location of POM-AMB-acy in U251 cells, scanning transmission electron microscopy (STEM) was employed to observe the location of molybdenum, and energy dispersive X-Ray spectroscopy (EDX) was utilized to track the ratio of molybdenum in each area. As shown in Fig. 4a, bright spots were clearly located inside the U251 cell and the ratio of molybdenum in this area reached 2.83% compared with the untreated cell (nearly 0%). Moreover, since the shading degree can indicate the concentration of molybdenum in STEM, there was an extremely bright oval area comparing with surroundings and the ratio of molybdenum in this area significantly climbed to 8.92% (Fig. 4b). Furthermore, the penetrating ability of POM-AMB-acy towards BBB was evaluated. The Balb/c mice were treated with POM-AMB-acy *via* tail vein injection. As shown in Table S1, after dosing with POM-AMB-acy (12.5 mg kg^−1^) for 10 min, the molybdenum level in brain rapidly reached 4.4 mg kg^−1^, respectively. While in the control group, the molybdenum level was merely 0.2 mg kg^−1^.

### Degradability of POM-AMB-acy

**Figure 5:**
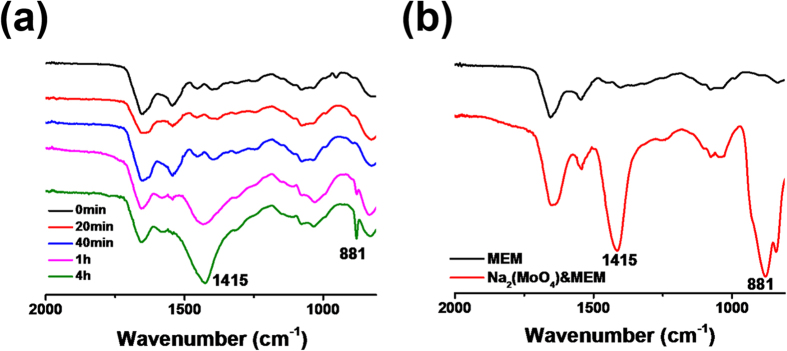
The stability of POM-AMB-acy in MEM solution. (**a**) Time dependent IR spectrum of POM-AMB-acy in MEM solution. (**b**) The IR spectrum of MEM solution and Na(MoO_4_)_2_ pretreated with MEM for 2 hours.

Apart from the above characteristics, the degradability of POM-AMB-acy is of great importance. In order to prove this point, *in vitro* experiments were performed to investigate its degradability by utilizing the fresh cell incubation medium (MEM + 10% FBS) as demonstration. In order to investigate its stability in MEM, IR and ESI-MS was chosen to study its stability. According to Fig. 5a, POM-AMB-acy stayed intact in MEM within 40 min. While after 1 hour’s treatment with MEM, new bands appeared at 1415 and 881 cm^−1^ compared with 40 min, and it can also be seen in the spectrum of Na_2_MoO_4_ in MEM (Fig. 5b). Moreover, according to Figure S6, the time dependent IR spectra for another organoimido-derivatized POMs (POM-Ad) indicated that admantadine modified POM cannot be degraded into MoO_4_^2−^ in MEM which furthermore proved that the optimized ligand was sufficient for POM’s degradability.

Furthermore, the ESI-MS was also conducted to investigate the composition of degradation product. After maintaining the system (solute: POM-AMB-acy, solvent: MEM + 10% FBS) for 24 h, this inorganic degraded product was marked as complex **T1**. As shown in Figure S2, the ESI-MS spectrum for **T1** was quite different from that for POM-AMB-acy itself. The characteristic peaks in POM-AMB-acy are located at 609.28, 1219.58 and 1460.86 (Figure S1). However, the major peaks for **T1** are located at 80.95, 159.95 and 198.03, which are in accordance with the characteristic peaks for [Bu_4_N]_2_[MoO_4_] (calcd m/z: 79.97, 159.94 and 200.7). Recent researches have reported that molybdenum oxide (MoOx) nanosheets could also be degraded into MoO_4_^2−^ in serum, which is corresponding to our result^43^. Therefore, in the cell incubation environment, POM-AMB-acy can be ultimately degraded into MoO_4_^2−^.

### Cytotoxicity of POM-AMB-acy

**Figure 6:**
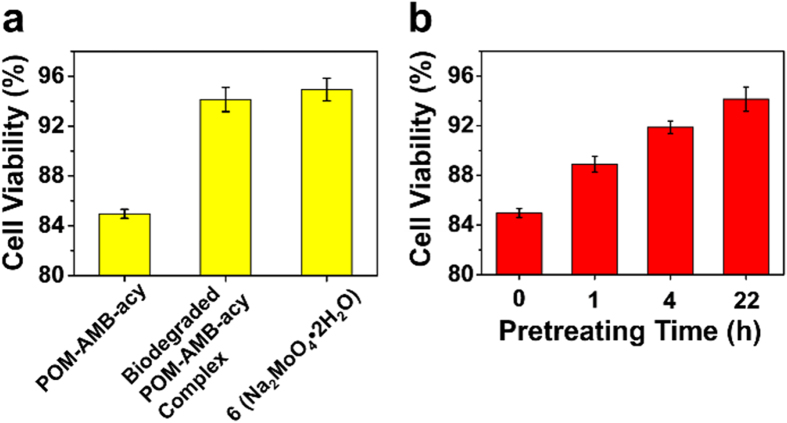
The effect of degradation on the cytotoxicity of POMs. (**a**) Cell viability of PC12 cells by incubating with POM-AMB-acy and its related complex under the concentration of 40 μM. (**b**) The relationship between pretreating time and viability of PC12 cell.

To understand whether the degradable characteristic is critical to alleviating the toxicity of POM-AMB-acy, POM-AMB-acy itself and degraded POM-AMB-acy complex (**T1**) on cellular metabolism behavior towards PC12 cell was explored. As shown in Fig. 6a, under the concentration of 40 μM, the cell viability for POM-AMB-acy was around 84.9% while that for degraded POM-AMB-acy complex can be improved to 94.1% which is comparative with ‘6 equals of Na_2_MoO_4_·2 H_2_O’ (94.9%). Therefore, it is deduced that the degradability could alleviate the toxicity of POM-AMB-acy on certain degree. To further investigate the dynamics of degrading process on cytotoxicity of PC12 cells, POM-AMB-acy was pretreated with the cell incubation medium (MEM + 10% FBS) for certain hours, including 0 h, 1 h, 4 h and 22 h, and then added into PC12 cells for further incubation. As shown in Fig. 6b, when the pretreatment was performed for 1 h before POM-AMB-acy being added into PC12 cells, the cell viability of it was higher than original POM-AMB-acy with 5% increase. Moreover, after 4 hours’ pretreatment, the cell viability reached 91.9%, which was close to the degraded complex (**T1**). Therefore, POM-AMB-acy can be degraded and achieved low toxicity no more than 22 hours. Therefore, via degradation, the toxicity of POM-AMB-acy can be reduced and the long-term toxicity of POM can be alleviated on a certain degree.

## Discussion

In conclusion, a degradable organically-derivatized POM was developed with high efficacy towards glioblastoma cancer cell. To prevent the side effects, this agent is endowed with degradability by introducing a cleavable functional group into its structure. This fundamental research can provide the guidance to fabricate other degradable agents based on POMs or nanoclusters for cancer therapy. All in all, this line of research represents a great demonstration as a POM-based compound for glioblastoma inhibition and provides an effective approach to solve the problems in the medicinal chemistry of POMs. This research will enrich the field of glioblastoma inhibition and medicinal chemistry of POMs with important advances.

## Methods

### Materials

All chemicals were purchased and used as supplied without further purification. Acetonitrile was distilled by refluxing in the presence of CaH_2_ overnight. [Bu_4_N]_2_[Mo_6_O_19_] was prepared by the treatment of Na_2_MoO_4_·2H_2_O with HCl and tetrabutylammonium bromide in water, according to literature methods^44^. Other chemical reagents used in the synthesis were analytical pure and without further purification. Elemental analyses were performed on a Flash EA 1112 full-automatic microanalyser.

### Spectroscopic Characterization

IR spectra were measured by using a Perkin Elmer FT-IR spectrophotometer on KBr pellets in the range of 4000–400 cm^−1^ with the resolution of 4 cm^−1^. Relative intensities are given after the wavenumber as vs = very strong, s = strong, m = medium, w = weak, sh. = shoulder, br. = broad. ESI-MS spectra were obtained by using a Finnigan LCQ Deca XP Plus ion trap mass spectrometer (San Jose, CA), and all experiments were carried out in the negative-ion mode.

### Crystallographic structural determinations

A red single crystal of POM-AMB-acy with three dimensions of 0.40 mm × 0.50 mm × 0.50 mm was selected for diffraction analysis. The data collection was performed on a Rigaku RAXIS-SPIDER IP diffractometer at 50 kV and 20 mA, using graphite monochromatized Mo *K*_α_ radiation (λ = 0.71073 Å) at 94(2) K. Data collection, data reduction, cell refinement, and experimental absorption correction were performed with the software package of Rigaku RAPID AUTO (Rigaku, 1998, Ver2.30). Structures were solved by direct methods and refined against F^2^ by full matrix least squares. All non-hydrogen atoms, except disordered atoms, were refined anisotropically. Hydrogen atoms were generated geometrically. All calculations were performed using the SHELXS-97 program package.

### Cell culture

U251 (human malignant glioblastoma) and PC12 cells (rat pheochromocytoma) were obtained from the Cancer Institute of Chinese Academy of Medical Science (Beijing, China) and grown in MEM supplemented with 5% fetal bovine serum and 10% horse serum in a humidified 5% CO_2_ environment at 37.0 °C. Cells were plated at a density of 1 × 10^6^ cells per 100 mm culture dish and allowed to grow to approximately 70% confluence before experimentation.

### Scanning transmission electron microscopy (STEM) and Energy Dispersive X-ray Spectrum (EDX)

U251 cells were seeded in 10 cm dishes at a density of 1 × 10^5^ cells/mL. After 24 h incubation, cells were treated with **1** at the concentration of 60 μM for 24 h. The cells were directly harvested with a cell scraper and centrifuged at 2500 rpm for 10 min. After fixation by a 2.5% (wt/vol) glutaraldehyde and 2% (wt/vol) paraformaldehyde, samples were then submitted to the Center of Biomedical Analysis (Tsinghua University) for subsequent treatment and sent to Analysis and Test Center (Tsinghua University) for STEM and EDX analysis. Of note, in order to clearly identify the distribution of molybdenum in cancer cells using STEM mode, it had to give up using osmium tetroxide for fixation, so the morphology of U251 cells lost on a certain degree.

### Synthesis of POM-Ad

According to the previous literature^16^, a mixture of (n-Bu_4_N)_4_[Mo_8_O_26_] (1.5 mmol, 3.23 g), amantadine hydrochloride (2 mmol, 0.38 g) and DCC (3 mmol, 0.62 g) were added into 10 mL anhydrous acetonitrile and refluxed under dry N_2_ for 9 hours. During the reaction procedure, reactants gradually dissolved and the color of the solution turned into light green. By cooling it down to room temperature, the white precipitate (N,N’-dicyclohexylurea) was moved by filtration. With the slow evaporation of acetonitrile from the filtrate, the yellow block crystals appeared (1.93 g, yield 61%). Elemental analysis Calc (%) for C_46_H_93_Mo_6_N_5_O_18_ (M = 1579.89): C, 34.94; N, 4.43; H, 5.89. Found: C, 34.88; N, 4.39; H, 5.85. IR (KBr pellet, major peaks, cm^−1^): 2961, 2932, 2874, 1481, 1380, 1236, 973, 944, 783 (absorbance at 973 is characteristic peak for mono-organoimido substituted hexamolybdate). UV/vis (MeCN, nm): λ_max_ = 325. ESI-mass spectrometry (MeCN, m/z): 1254.7 (calculated 1255.1), 1015.4 (calculated 1013.6), and 508.2 (calculated 506.3) were assigned to [Bu_4_N][Mo_6_O_18_N(C_10_H_15_)]^−^, [HMo_6_O_18_N(C_10_H_15_)]^−^ and [Mo_6_O_18_N(C_10_H_15_)]^2−^, respectively.

### Synthesis of POM-AMB-acy

A mixture of (Bu_4_N)_2_[Mo_6_O_19_] (2 mmol, 2.73 g), 2-amino-3-methylbenzoic acid (2 mmol, 0.30 g) and DCC (2.2 mmol, 0.45 g) were added into 15 mL anhydrous acetonitrile at 110 °C under the protection of dry N_2_. During the reaction, the color of the solution gradually changed from orange to red then the dark red. After 20 hours, by cooling to room temperature, solution was filtrated to remove the white precipitate (*N, N’*-dicyclohexylurea). Slowly evaporating the filtrate in open air, some sticky oil was obtained. Then it was carefully washed by toluene and ether for several times. By the slow gas-phase diffusion of ether into the filtrate, POM-AMB-acy was deposited as red crystals within several days. Yield of (Bu_4_N)_2_[Mo_6_O_18_(≡NC_6_H_4_-2-CH_3_-6-CON(Cy)-CO-NH-Cy)]: 0.78 g, 23%, based on Mo. Elemental analysis (%) calcd for Mo_6_O_20_C_53_N_5_H_102_ (M = 1705.05 g mol^−1^): C 37.33, N 4.11, H 5.98; found: C 37.40, N 4.08, H 5.98; IR (KBr pellet, major peaks, cm^−1^): 2961 (m), 2933 (m), 2874 (w), 1482 (w), 1380 (w), 976 (sh), 951 (sh), 783 (sh) (the band at 976 is a characteristic peak for mono-organoimido-substituted hexamolybdate); UV/vis (MeCN, nm): λ_max_ = 351; ESI-MS (MeCN, m/z): 1460.86 (calcd 1461.6), 1219.58 (calcd 1220.1), and 609.28 (calcd 609.56), assigned to [Bu_4_N][Mo_6_O_18_(≡NC_6_H_4_-2-CH_3_-6-CON(Cy)-CO-NH-Cy)]^−^, [HMo_6_O_18_(≡NC_6_H_4_-2-CH_3_-6-CON(Cy)-CO-NH-Cy)]^−^, and [Mo_6_O_18_(≡NC_6_H_4_-2-CH_3_-6-CON(Cy)-CO-NH-Cy)]^2−^, respectively.

Crystal data for compound (POM-AMB-acy)(C_4_H_10_O): Mo_6_O_21_C_57_N_5_H_110_, M = 1777.14, Monoclinic, space group *P*2_1/c_, *a* = 11.7348(14) Å, *b* = 25.846(2) Å, *c* = 23.951(3) Å, *α* = 90°, *β* = 93.220(13)°, *γ* = 90°, *V* = 7252.8(14) Å^3^, *T* = 94 K, *Z* = 4, *D*c = 1.628 g cm^−3^, *m* = 1.076 mm^−1^, GooF = 1.088, Final *R* indices (I ≥ 2σ(I)) *R*_1_ = 0.0755, *wR*_2_ = 0.1559.

### Degrading process on cytotoxicity assays

To evaluate the cytotoxicity of degraded POM-AMB-acy complex, the PC12 cells were seeded at density of 5 × 10^4^ cells per well in a 96-well microtiter plate in advance. To prepare the degraded POM-AMB-acy complex, POM-AMB-acy was pretreated with 100 μL of mixture solution (10 μL of DMSO solution and 90 μL of cell medium) for corresponding period (0 h, 1 h, 4 h and 22 h) at the concentration of 80 μM. After 24 h of incubation, the cells were treated with 100 μL of cell medium and 100 μL of mixture solution which contains the pretreated POM-AMB-acy, so the final concentration of POM-AMB-acy was 40 μM and the cells were incubated for another 24 h. For each test, five replicates were employed. Twenty microliters of 5 mg/mL MTT solution were added to each well, and cells continued to be incubated for 4 h at 37.0 °C. After careful removal of the medium, dimethyl sulfoxide (DMSO) was added to each well, and the plate was then shaken for about 10 min. Absorbance was then measured at 490 nm in a microplate reader (Scientific Varioskan Flash, Thermo Fisher Scientific, U.S.A). The curves of viability were drawn by comparing the control group. The inhibitory rate was calculated using the following equation: inhibitory rate (%) = (OD_control_ − OD_treatment_)/OD_control_ × 100%. The viability rate (%) = 100 − inhibitory rate (%).

## Additional Information

**How to cite this article**: She, S. *et al.* Degradable Organically-Derivatized Polyoxometalate with Enhanced Activity against Glioblastoma Cell Line. *Sci. Rep.* **6**, 33529; doi: 10.1038/srep33529 (2016).

## Supplementary Material

Supplementary Information

Supplementary Dataset 1

Supplementary Dataset 2

## Figures and Tables

**Figure 1:**
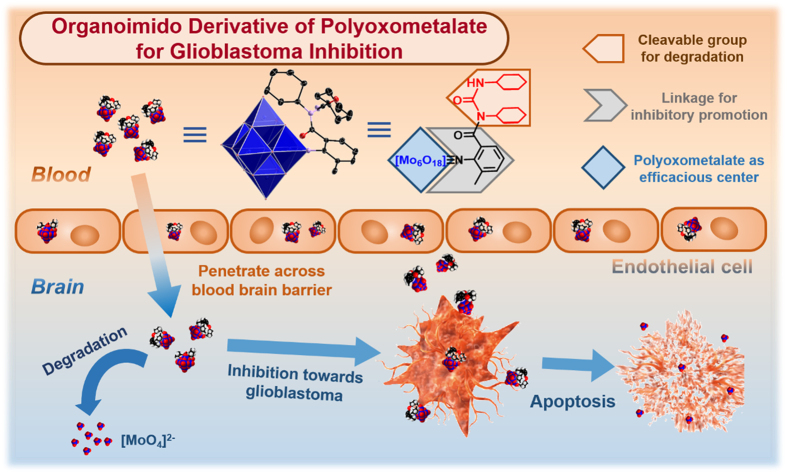
Schematic representation of the overall process of POM-AMB-acy for glioblastoma inhibition. The design of POM-AMB-acy and the overall process of blood brain barrier penetration, inhibition toward malignant glioma cell (U251) and degradation.

**Table 1:** IC_50_ Values of POM-AMB-acy and its comparative complexes towards malignant glioma cell U251.

Sample	IC_50_^a^ (μM)
POM-AMB-acy	24.8 ± 0.3
POM-Ad	31.1 ± 0.3
[Bu_4_N]_2_[Mo_6_O_19_]	32.4 ± 0.3
6 equals of Na_2_MoO_4_•2 H_2_O	53.4 ± 0.2
TMZ^29^	< 500

^a^An MTT assay was used to evaluate the average IC_50_ value using five independent experiments. The mean values of five measurements are shown.
