# Supplementary material for: Degradable Organically-Derivatized Polyoxometalate with Enhanced Activity against Glioblastoma Cell Line
Source: Sci Rep. 2016 Sep 23;6:33529. doi: 10.1038/srep33529 (PMC5034237; doi:10.1038/srep33529)
Supplement: Supplementary Information [file srep33529-s1.doc]

Degradable Organically-Derivatized Polyoxometalate with Enhanced Activity against Glioblastoma Cell Line

Shan She, Shengtai Bian, Ruichao Huo, Kun Chen,^*^ Zehuan Huang, Jiangwei Zhang, Jian Hao,^*^ and Yongge Wei^*^

**Contents**

1. Materials and Methods.
2. Figure S1. ESI-MS of POM-AMB-acy.
3. Figure S2. ESI-MS of complex **T1**.
4. Figure S3. Analysis of inhibition effect of POM-AMB-acy towards U251 cell by MTT assay.
5. Figure S4. STEM and EDX result of POM-AMB-acy towards U251 cell.
6. Figure S5. ESI-MS of (Bu_4_N)_2_[MoO_4_].
7. Figure S6. Time independent IR spectrum of POM-Ad treated with MEM.
8. The possible degrade process of POM-AMB-acy.
9. Table S1. Blood-brain barrier penetration assay.
10. References.
11. **Materials and Methods.**

**General Remarks.** All chemicals were purchased and used as supplied without further purification. Acetonitrile was distilled by refluxing in the presence of CaH_2_ overnight. [Bu_4_N]_2_[Mo_6_O_19_] was prepared by the treatment of Na_2_MoO_4_·2H_2_O with HCl and tetrabutylammonium bromide in water, according to literature methods. ^[1]^ Other chemical reagents used in the synthesis were analytical pure and without further purification. Elemental analyses were performed on a Flash EA 1112 full-automatic microanalyser.

**Spectroscopy.** IR spectra were measured by using a Perkin Elmer FT-IR spectrophotometer on KBr pellets in the range of 4000-400 cm^-1^ with the resolution of 4 cm^-1^. Relative intensities are given after the wavenumber as vs = very strong, s = strong, m = medium, w = weak, sh. = shoulder, br. = broad. ESI-MS spectra were obtained by using a Finnigan LCQ Deca XP Plus ion trap mass spectrometer (San Jose, CA), and all experiments were carried out in the negative-ion mode.

**X-ray Crystallography.** A red single crystal of POM-AMB-acy with three dimensions of 0.40 mm × 0.50 mm × 0.50 mm was selected for diffraction analysis. The data collection was performed on a Rigaku RAXIS-SPIDER IP diffractometer at 50 kV and 20 mA, using graphite monochromatized Mo *K*_α_ radiation (λ = 0.71073 Å) at 94(2) K. Data collection, data reduction, cell refinement, and experimental absorption correction were performed with the software package of Rigaku RAPID AUTO (Rigaku, 1998, Ver2.30). Structures were solved by direct methods and refined against F^2^ by full matrix least squares. All non-hydrogen atoms, except disordered atoms, were refined anisotropically. Hydrogen atoms were generated geometrically. All calculations were performed using the SHELXS-97 program package.

**Cell culture.** U251 (human malignant glioblastoma) and PC12 cells (rat pheochromocytoma) were obtained from the Cancer Institute of Chinese Academy of Medical Science (Beijing, China) and grown in MEM supplemented with 5 % fetal bovine serum and 10 % horse serum in a humidified 5 % CO_2_ environment at 37.0 °C. Cells were plated at a density of 1 x 10^6^ cells per 100 mm culture dish and allowed to grow to approximately 70 % confluence before experimentation.

**Cell proliferation and cytotoxicity assays.** Cells were seeded in a 96-well microtiter plate (Nunc, Wiesbaden, Germany) at 1 x 10^4^ cells (U251) per well. After 24 h of incubation, the cells were treated with 200 μL solution (10 μL of DMSO solution and 190 μL of cell medium) containing certain concentrations (5 μM, 10 μM, 20 μM, 30 μM, 40 μM, 60 μM) of corresponding compounds (Na_2_MoO_4_, [Bu_4_N]_2_[Mo_6_O_19_] and POM-AMB-acy) for 24 h. For each concentration, five replicates were employed. Twenty microliters of 5 mg/mL MTT solution were added to each well, and cells continued to be incubated for 4 h at 37.0 °C. After careful removal of the medium, dimethyl sulfoxide (DMSO) was added to each well, and the plate was then shaken for about 10 min. Absorbance was then measured at 490 nm in a microplate reader (Scientific Varioskan Flash, Thermo Fisher Scientific, U.S.A). The curves of viability were drawn by comparing the control group. The inhibitory rate was calculated using the following equation: inhibitory rate (%) = (OD_control_ - OD_treatment_) / OD_control_×100 %.

As for the cytotoxicity assays, cells were seeded in a 96-well microtiter plate (Nunc, Wiesbaden, Germany) at 5 x 10^4^ cells (PC12) per well. After 24 h of incubation, the cells were treated with 200 μL solution (10 μL of DMSO solution and 190 μL of cell medium) containing 40 μM of corresponding compounds (Na_2_MoO_4_, POM-AMB-acy and degraded POM-AMB-acy complex) for 24 h. For each concentration, five replicates were employed. Twenty microliters of 5 mg/mL MTT solution were added to each well, and cells continued to be incubated for 4 h at 37.0 °C. After careful removal of the medium, dimethyl sulfoxide (DMSO) was added to each well, and the plate was then shaken for about 10 min. Absorbance was then measured at 490 nm in a microplate reader (Scientific Varioskan Flash, Thermo Fisher Scientific, U.S.A). The curves of viability were drawn by comparing the control group. The inhibitory rate was calculated using the following equation: inhibitory rate (%) = (OD_control_ - OD_treatment_) / OD_control_×100 %. The viability rate (%) = 100 - inhibitory rate (%).

**Degrading process on cytotoxicity assays.** To evaluate the cytotoxicity of degraded POM-AMB-acy complex, the PC12 cells were seeded at density of 5 x 10^4^ cells per well in a 96-well microtiter plate in advance. To prepare the degraded POM-AMB-acy complex, POM-AMB-acy was pretreated with 100 μL of mixture solution (10 μL of DMSO solution and 90 μL of cell medium) for corresponding period (0 h, 1 h, 4 h and 22 h) at the concentration of 80 μM. After 24 h of incubation, the cells were treated with 100 μL of cell medium and 100 μL of mixture solution which contains the pretreated POM-AMB-acy, so the final concentration of POM-AMB-acy was 40 μM and incubated for another 24 h. For each one, five replicates were employed. Twenty microliters of 5 mg/mL MTT solution were added to each well, and cells continued to be incubated for 4 h at 37.0 °C. After careful removal of the medium, dimethyl sulfoxide (DMSO) was added to each well, and the plate was then shaken for about 10 min. Absorbance was then measured at 490 nm in a microplate reader (Scientific Varioskan Flash, Thermo Fisher Scientific, U.S.A). The curves of viability were drawn by comparing the control group. The inhibitory rate was calculated using the following equation: inhibitory rate (%) = (OD_control_ - OD_treatment_) / OD_control_×100 %. The viability rate (%) = 100 - inhibitory rate (%).

**Synthesis of POM-AMB-acy**

A mixture of (Bu_4_N)_2_[Mo_6_O_19_] (2 mmol, 2.73 g), 2-amino-3-methylbenzoic acid (2 mmol, 0.30 g) and DCC (2.2 mmol, 0.45 g) were added into 15 mL anhydrous acetonitrile at 110 °C under the protection of dry N_2_. During the reaction, the color of the solution gradually changed from orange to red then the dark red. After 20 hours, by cooling to room temperature, solution was filtrated to remove the white precipitate (*N, N’*-dicyclohexylurea). Slowly evaporating the filtrate in open air, some sticky oil was obtained. Then it was carefully washed by toluene and ether for several times. By the slow gas-phase diffusion of ether into the filtrate, POM-AMB-acy was deposited as red crystals within several days. Yield of (Bu_4_N)_2_[Mo_6_O_18_(≡NC_6_H_4_-2-CH_3_-6-CON(Cy)-CO-NH-Cy)]: 0.78 g, 23 %, based on Mo. Elemental analysis (%) calcd for Mo_6_O_20_C_53_N_5_H_102_ (M=1705.05 g mol^-1^): C 37.33, N 4.11, H 5.98; found: C 37.40, N 4.08, H 5.98; IR (KBr pellet, major peaks, cm^-1^): 2961 (m), 2933 (m), 2874 (w), 1482 (w), 1380 (w), 976 (sh), 951 (sh), 783 (sh) (the band at 976 is characteristic peak for mono-organoimido-substituted hexamolybdate); UV/Vis (MeCN, nm): λ_max_ = 351; ESI-MS (MeCN, m/z): 1460.86 (calcd 1461.6), 1219.58 (calcd 1220.1), and 609.28 (calcd 609.56), assigned to [Bu_4_N][Mo_6_O_18_(≡NC_6_H_4_-2-CH_3_-6-CON(Cy)-CO-NH-Cy)]^-^, [HMo_6_O_18_(≡NC_6_H_4_-2-CH_3_-6-CON(Cy)-CO-NH-Cy)]^-^, and [Mo_6_O_18_(≡NC_6_H_4_-2-CH_3_-6-CON(Cy)-CO-NH-Cy)]^2-^, respectively.

**Synthesis of POM-Ad**

According to the previous literature, a mixture of (n-Bu_4_N)_4_[Mo_8_O_26_] (1.5 mmol, 3.23 g), amantadine hydrochloride (2 mmol, 0.38 g) and DCC (3 mmol, 0.62 g) were added into 10mL anhydrous acetonitrile and refluxed under dry N_2_ for 9 hours. During the reaction procedure, reactants gradually dissolved and the color of the solution turned into light green. By cooling it down to room temperature, the white precipitate (N,N’-dicyclohexylurea) was moved by filtration. With the slow evaporation of acetonitrile from the filtrate, the yellow block crystals appeared (1.93 g, yield 61%). Elemental analysis Calc (%) for C_46_H_93_Mo_6_N_5_O_18_ (M= 1579.89): C, 34.94; N, 4.43; H, 5.89. Found: C, 34.88; N, 4.39; H, 5.85. IR (KBr pellet, major peaks, cm^-1^): 2961, 2932, 2874, 1481, 1380, 1236, 973, 944, 783 (absorbance at 973 is characteristic peak for mono-organoimido substituted hexamolybdate). UV/vis (MeCN, nm): λ_max_ = 325. ESI-mass spectrometry (MeCN, m/z): 1254.7 (calculated 1255.1), 1015.4 (calculated 1013.6), and 508.2 (calculated 506.3) were assigned to [Bu_4_N][Mo_6_O_18_N(C_10_H_15_)]^-^, [HMo_6_O_18_N(C_10_H_15_)]^-^ and [Mo_6_O_18_N(C_10_H_15_)]^2-^, respectively.

1. **Figure S1.**


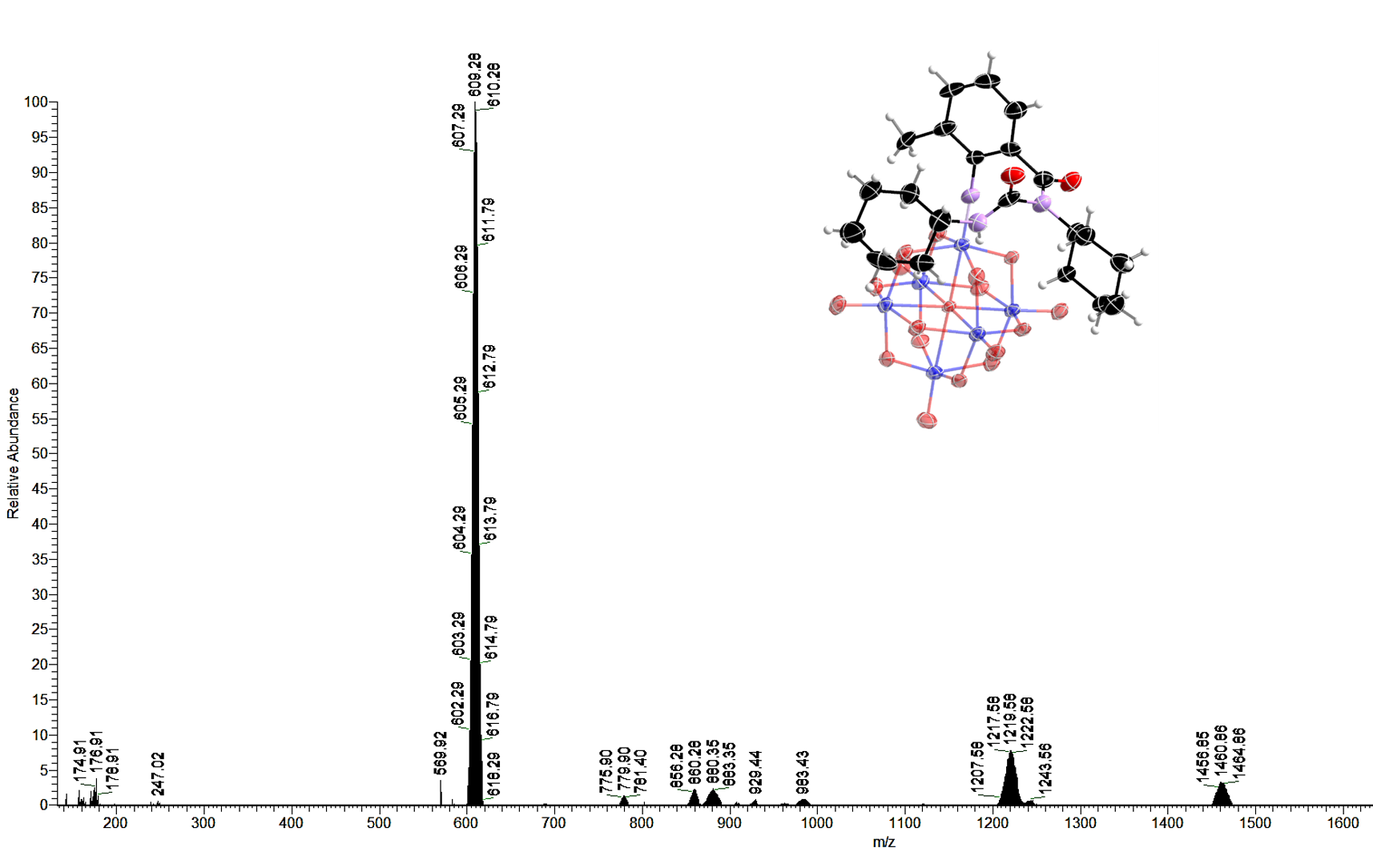


**Figure S1.** ESI-MS of POM-AMB-acy.

1. **Figure S2.**


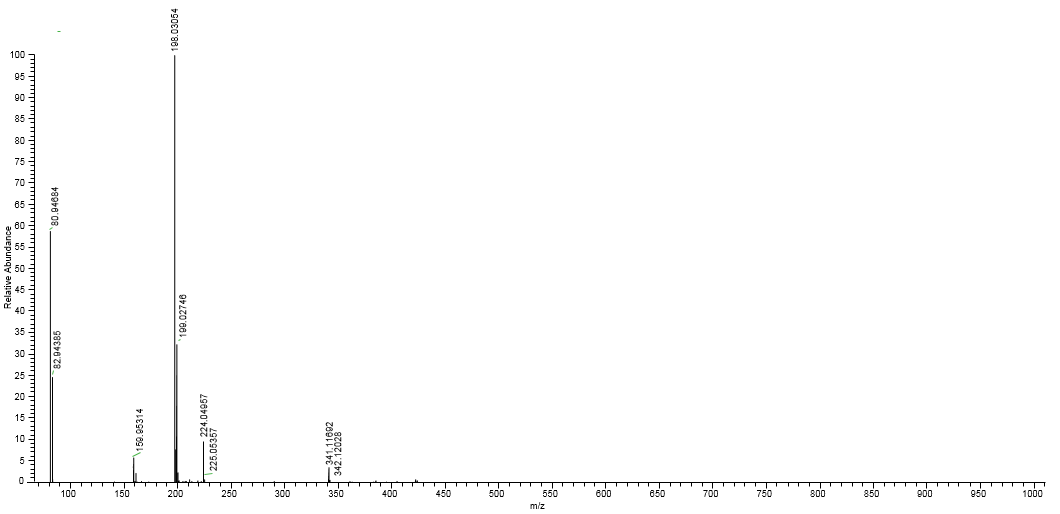


**Figure S2.** ESI-MS of complex **T1**.

1. **Figure S3.**

**
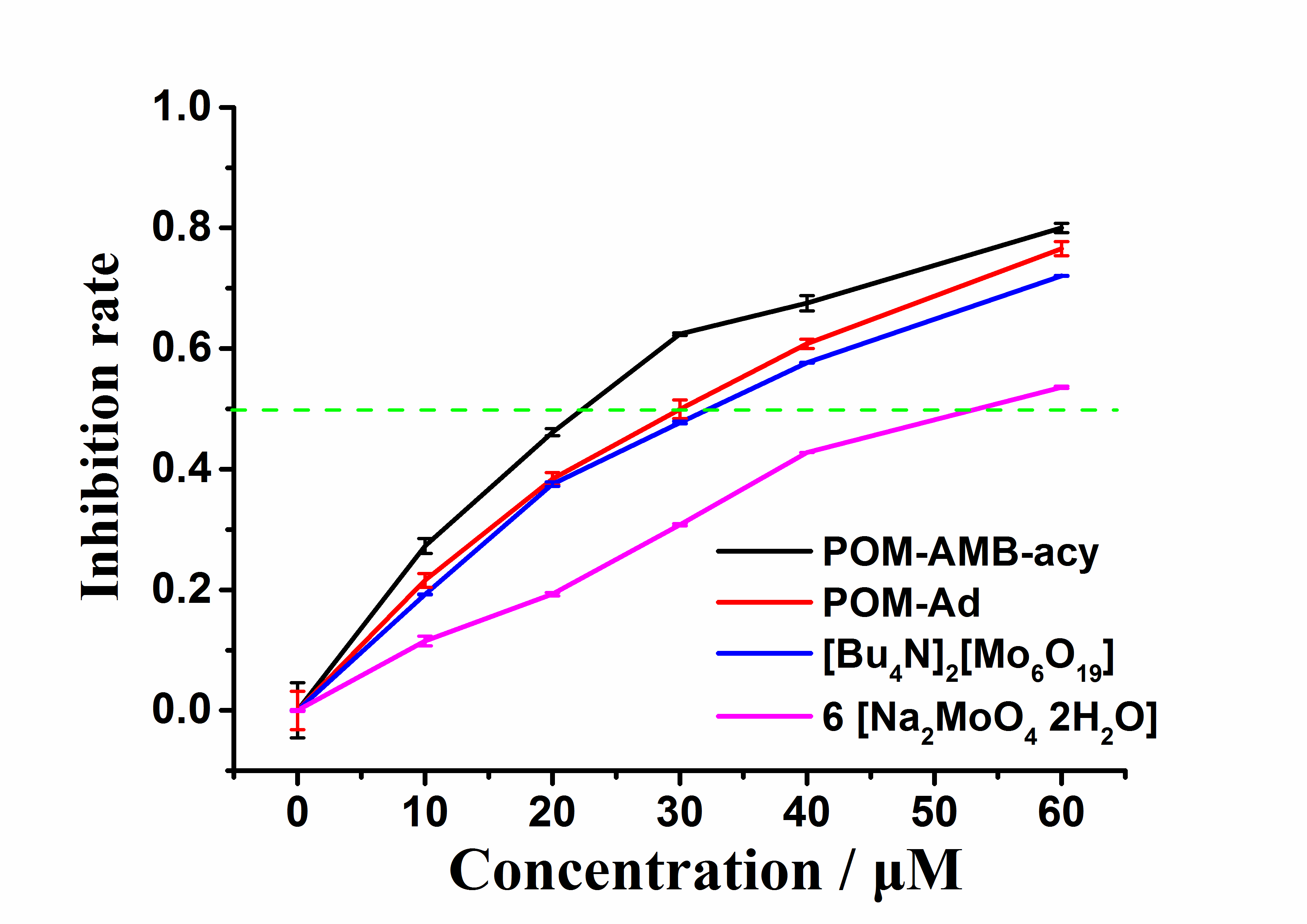
**

**Figure S3.** Analysis of inhibition effect by MTT assay.

1. **Figure S4.**


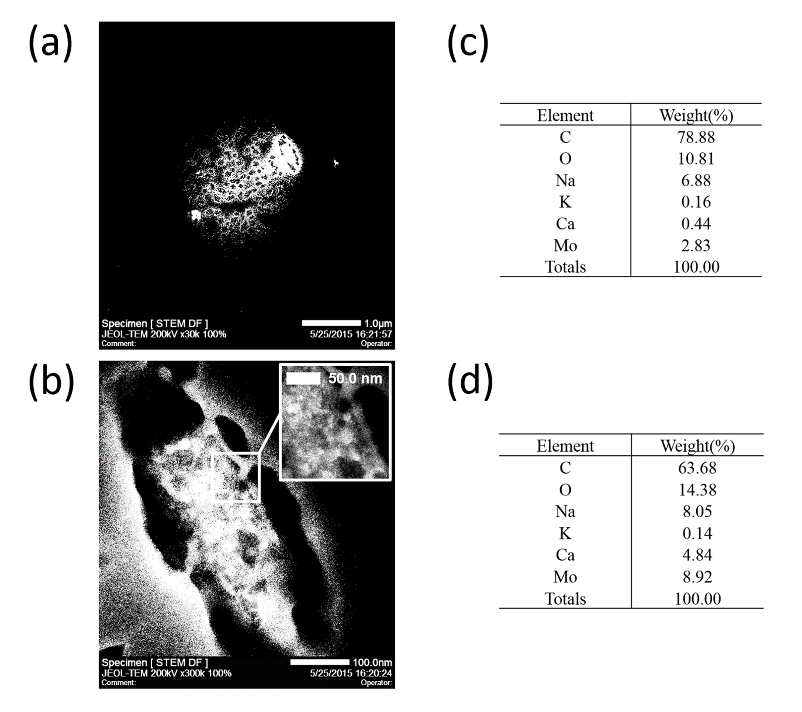


**Figure S4. STEM and EDX result of POM-AMB-acy towards U251 cell.** (a) Observation of molybdenum distribution in U251 cells infected with POM-AMB-acy by high-resolution transmission EM (STEM mode). Scale bar = 1 μm. (b) Higher magnification of the upper site which was shinny bright in Figure S5a, Scale bar = 100 nm. The box in top (Scale bar = 50 nm) showed the magnification of the outlined in white area in Figure S5b and indicated that molybdenum based complex tend to form agglomerate. (c) EDX spectrum of Figure S5a. (d) EDX spectrum of outlined in white area in Figure S5b.

1. **Figure S5.**


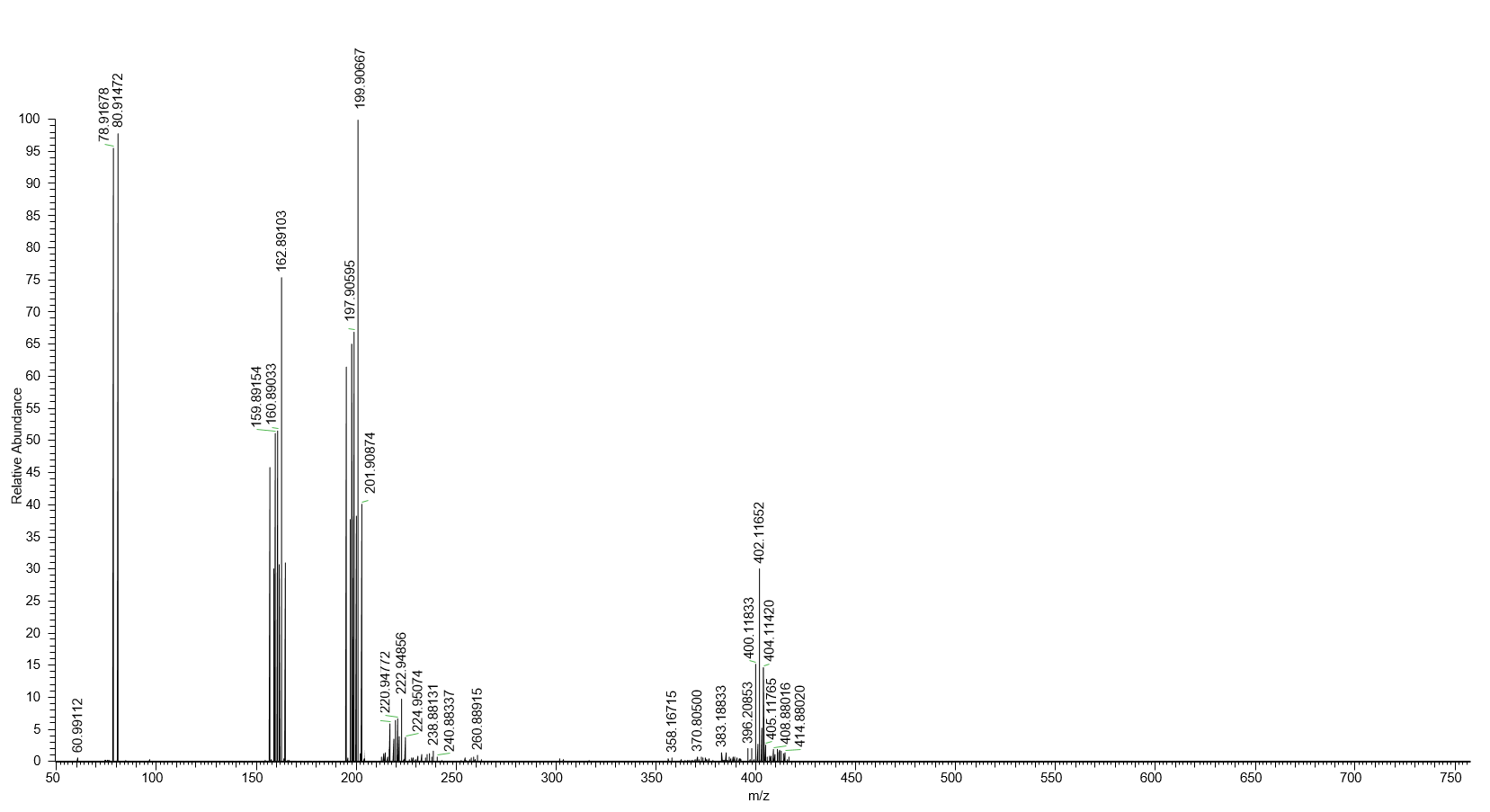


**Figure S5.** ESI-MS of (Bu_4_N)_2_[MoO_4_].

1. **Figure S6.**

**Figure S6.** Time independent IR spectrum of POM-Ad treated with MEM.

1. **The possible degrade process of POM-AMB-acy.**

1. **Table S1.**

**Table S1.** Blood-brain barrier penetration assay

| **Sample** | | **Concentration of Mo^[a]^** |
| --- | --- | --- |
| Brain | Control | 0.2 mg kg^-1^ |
|  | dosing with **POM-AMB-acy** (12.5 mg kg^-1^) | 4.4 mg kg^-1^ |
| [a] The plasma and brain homogenates were analyzed for levels of molybdenum by inductively coupled plasma mass spectrometry (ICP-MS) | | |

1. **Reference**

[1] N. Hur, W. Klemperer, R. Wang, M. Fournier, P. Alvin*, Inorg. Synt***h 19**90*,* 27, 77-78.
