# Supplementary material for: Degradable Organically-Derivatized Polyoxometalate with Enhanced Activity against Glioblastoma Cell Line
Source: Sci Rep. 2016 Sep 23;6:33529. doi: 10.1038/srep33529 (PMC5034237; doi:10.1038/srep33529)

checkCIF/PLATON report

No syntax errors found. **CIF dictionary** **Interpreting this report**

Datablock: compound1

Bond precision: C-C = 0.0162 A Wavelength=0.71073

**Cell:**	**a=11.7348(14)**	**b=25.846(2)**	**c=23.951(3)**
	**alpha=90**	**beta=93.220(13)**	**gamma=90**
**Temperature:**	**94 K**		

	Calculated	Reported
Volume	7252.8(14)	7252.8(14)
Space group	P 21/c	P 1 21/c 1
Hall group	-P 2ybc	-P 2ybc
Moiety formula	C21 H28 Mo6 N3 O20, 2(C16 H36 N), C4 H10 O	C21 H28 Mo6 N3 O20, 2(C16 H36 N), C4 H10 O
Sum formula	C57 H110 Mo6 N5 O21	C57 H110 Mo6 N5 O21
Mr	1777.14	1777.14
Dx,g cm-3	1.628	1.628
Z	4	4
Mu (mm-1)	1.076	1.076
F000	3628.0	3628.0
F000’	3587.99	
h,k,lmax	14,31,29	14,31,29
Nref	14255	14218
Tmin,Tmax	0.590,0.650	0.615,0.673
Tmin’	0.578	

Correction method= # Reported T Limits: Tmin=0.615 Tmax=0.673
AbsCorr = MULTI-SCAN

Data completeness= 0.997 Theta(max)= 26.000

R(reflections)= 0.0755(8779) wR2(reflections)= 0.1559(14218)

S = 1.088 Npar= 943

The following ALERTS were generated. Each ALERT has the format
test-name_ALERT_alert-type_alert-level.
Click on the hyperlinks for more details of the test.

Alert level B

PLAT413_ALERT_2_B Short Inter XH3 .. XHn H11K .. H22G .. 1.90 Ang.

Alert level C

```
PLAT165_ALERT_3_C Nr. of Status R Flagged Non-Hydrogen Atoms .....          5
PLAT220_ALERT_2_C Large Non-Solvent  C     Ueq(max)/Ueq(min) Range        3.4 Ratio
PLAT220_ALERT_2_C Large Non-Solvent  C     Ueq(max)/Ueq(min) Range        4.2 Ratio
PLAT222_ALERT_3_C Large Non-Solvent  H     Uiso(max)/Uiso(min) ...        4.3 Ratio
PLAT222_ALERT_3_C Large Non-Solvent  H     Uiso(max)/Uiso(min) ...        5.2 Ratio
PLAT234_ALERT_4_C Large Hirshfeld Difference C143   --  C144    ..       0.18 Ang.
PLAT244_ALERT_4_C Low   'Solvent' Ueq as Compared to Neighbors of         O21 Check
PLAT244_ALERT_4_C Low   'Solvent' Ueq as Compared to Neighbors of         C23 Check
PLAT250_ALERT_2_C Large U3/U1 Ratio for Average U(i,j) Tensor ....        2.7 Note
PLAT250_ALERT_2_C Large U3/U1 Ratio for Average U(i,j) Tensor ....        2.1 Note
PLAT342_ALERT_3_C Low Bond Precision on  C-C Bonds ...............     0.0162 Ang.
PLAT360_ALERT_2_C Short  C(sp3)-C(sp3) Bond  C22    -   C23    ...       1.40 Ang.
PLAT360_ALERT_2_C Short  C(sp3)-C(sp3) Bond  C24    -   C25    ...       1.41 Ang.
PLAT413_ALERT_2_C Short Inter XH3 .. XHn     H12K   ..  H24K    ..       2.12 Ang.
```

Alert level G

```
PLAT002_ALERT_2_G Number of Distance or Angle Restraints on AtSite          4 Note
PLAT003_ALERT_2_G Number of Uiso or Uij Restrained non-H Atoms ...         34 Report
PLAT005_ALERT_5_G No _iucr_refine_instructions_details  in the CIF     Please Do !
PLAT007_ALERT_5_G Number of Unrefined Donor-H Atoms ..............          1 Report
PLAT083_ALERT_2_G SHELXL Second Parameter in WGHT Unusually Large.      41.06 Why ?
PLAT301_ALERT_3_G Main Residue  Disorder ............ Percentage =         20 Note
PLAT304_ALERT_4_G Non-Integer Number of Atoms (  28.73) in Resd. #          3 Check
PLAT304_ALERT_4_G Non-Integer Number of Atoms (  24.27) in Resd. #          4 Check
PLAT343_ALERT_2_G Unusual sp?      Angle Range in Main Residue for     C4      Check
PLAT790_ALERT_4_G Centre of Gravity not Within Unit Cell: Resd.  #          2 Note
              C16 H36 N
PLAT860_ALERT_3_G Number of Least-Squares Restraints .............        507 Note
```

---

0 **ALERT level A** = Most likely a serious problem - resolve or explain
1 **ALERT level B** = A potentially serious problem, consider carefully
14 **ALERT level C** = Check. Ensure it is not caused by an omission or oversight
11 **ALERT level G** = General information/check it is not something unexpected

0 ALERT type 1 CIF construction/syntax error, inconsistent or missing data
12 ALERT type 2 Indicator that the structure model may be wrong or deficient
6 ALERT type 3 Indicator that the structure quality may be low
6 ALERT type 4 Improvement, methodology, query or suggestion
2 ALERT type 5 Informative message, check

It is advisable to attempt to resolve as many as possible of the alerts in all categories. Often the minor alerts point to easily fixed oversights, errors and omissions in your CIF or refinement strategy, so attention to these fine details can be worthwhile. In order to resolve some of the more serious problems it may be necessary to carry out additional measurements or structure refinements. However, the purpose of your study may justify the reported deviations and the more serious of these should normally be commented upon in the discussion or experimental section of a paper or in the "special_details" fields of the CIF. checkCIF was carefully designed to identify outliers and unusual parameters, but every test has its limitations and alerts that are not important in a particular case may appear. Conversely, the absence of alerts does not guarantee there are no aspects of the results needing attention. It is up to the individual to critically assess their own results and, if necessary, seek expert advice.

Publication of your CIF in IUCr journals

A basic structural check has been run on your CIF. These basic checks will be run on all CIFs submitted for publication in IUCr journals (*Acta Crystallographica, Journal of Applied Crystallography, Journal of Synchrotron Radiation*); however, if you intend to submit to *Acta Crystallographica Section C* or *E,* you should make sure that full publication checks are run on the final version of your CIF prior to submission.

Publication of your CIF in other journals

Please refer to the *Notes for Authors* of the relevant journal for any special instructions relating to CIF submission.

PLATON version of 21/04/2015; check.def file version of 09/03/2015

Datablock compound1 - ellipsoid plot

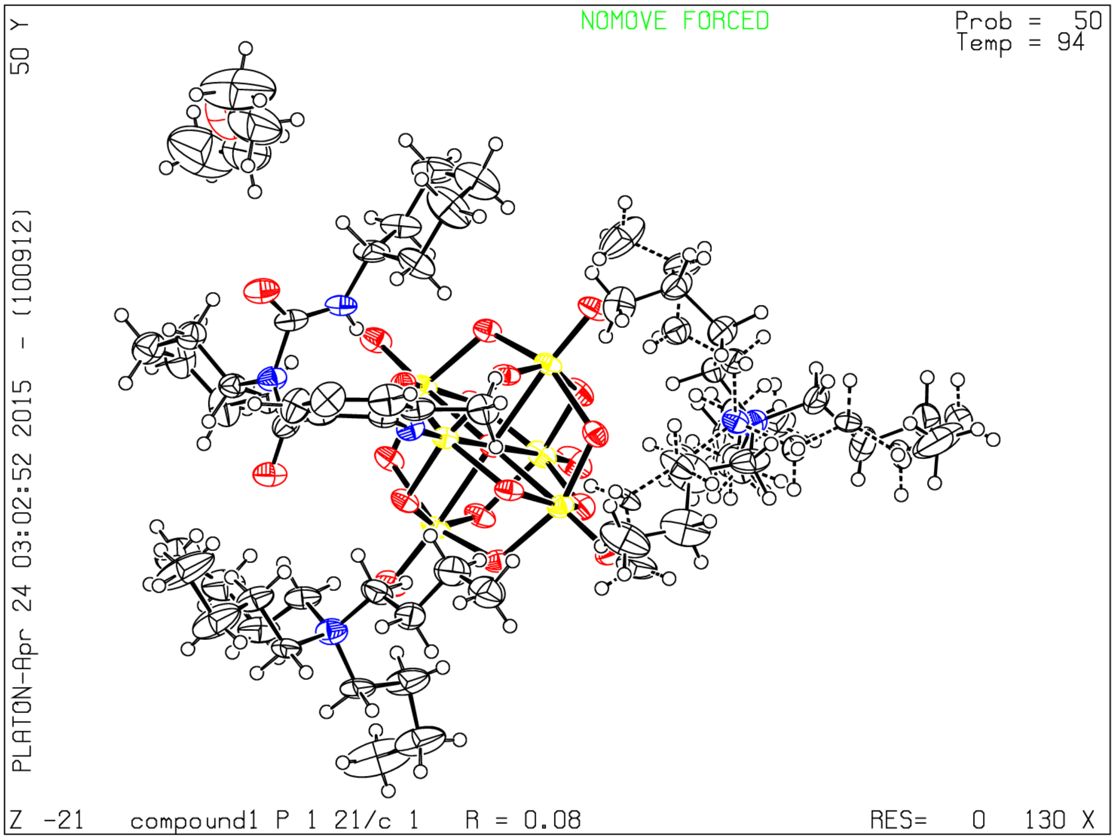

Supplement: Supplementary Dataset 1 [file srep33529-s2.zip › Checkcif Report for SP.pdf]
